# Assessment of Posterior Maxillary Alveolar Bone for Immediate Implant Placement: A Quantitative and Qualitative Analysis

**DOI:** 10.1055/s-0043-1777049

**Published:** 2024-02-08

**Authors:** Hussain M. Alkhames, Ramy Moustafa Moustafa Ali, Sukinah Sameer Alzouri, Mohamed Bayome

**Affiliations:** 1 Department of Prosthodontics and Dental Implantology, College of Dentistry, King Faisal University, Al-Ahsa, Saudi Arabia; 2 Department of Prosthodontics, Faculty of Dentistry, Fayoum University, Fayoum, Egypt; 3 Department of Restorative Dental Sciences, College of Dentistry, King Faisal University, Al-Ahsa, Saudi Arabia; 4 Department of Orthodontics, College of Dentistry, King Faisal University, Al-Ahsa, Saudi Arabia; 5 Department of Postgraduate Studies, Universidad Autonóma del Paraguay, Asunción, Paraguay

**Keywords:** immediate implant, maxillary bone, maxillary molars, bone height, bone density, CBCT

## Abstract

**Objectives** The aims of this study were to evaluate posterior maxillary alveolar bone dimensions and to compare these dimensions in males and females.

**Materials and Methods** The sample consisted of 102 cone beam computed tomography (CBCT) images for 62 male patients (mean age 29.92 ± 9.04 years) and 40 female patients (mean age 29.70 ± 9.54 years). Four distances and three densities were measured; a multivariate analysis of variance and Mann–Whitney's *U* test were applied to compare the differences between sexes.

**Results** For the first maxillary molar, there were significant differences between males and females in terms of coronal width (13.95 ± 1.31 and 13.22 ± 1.159 mm, respectively) and middle width (14.28 ± 1.43 and 13.57 ± 1.478 mm, respectively). However, no significant difference was found regarding height (7.93 ± 3.8 mm for both) or apical width (14.68 ± 2 mm for both). Regarding the second maxillary molar, significant differences between males and females were found in terms of coronal width (14.66 ± 1.63 and 13.54 ± 1.512 mm, respectively), middle width (14.35 ± 1.825 and 13.25 ± 1.52 mm, respectively), and height (7.29 ± 3.00 and 8.66 ± 3.16 mm, respectively), whereas the gender dimorphism regarding apical width had borderline significance (14.09 ± 1.731 mm; *p* = 0.048). No significant differences were found regarding density.

**Conclusion** The minimum average alveolar bone height for the second maxillary molar region was 7.29 ± 30 mm with significant gender dimorphism. Therefore, CBCT scans should be recommended prior to immediate implant placement.

## Introduction

Immediate implant placement refers to the placement of a dental implant immediately after the extraction of a tooth without the need for a healing period. This treatment option has gained popularity due to its high survival rate. 1 2 Immediate implant placement offers numerous advantages, including a reduction in the number of surgical procedures and treatment time. It also helps counteract the dimensional changes that occur in the socket after tooth extraction. 1 3

At the time of tooth extraction, the reparative process starts in the alveolar bone. Within 4 weeks after extraction, the socket will be filled with woven bone. 4 After that, this immature bone will be replaced with mature bone within 2 months. 4 However, during this process of remodeling and because of the lack of surrounding ligaments, the height of the buccal bone may undergo marked resorption. 5 In addition, during the process of bone healing, the bone width becomes diminished. 6 A systematic review showed that between 2.6 and 4.6 mm of the width of the bone becomes resorbed. 7 Moreover, a reduction ranging between 0.4 and 3.9 mm was observed in the height of the alveolar bone during healing after tooth extraction. 7 Furthermore, a prospective clinical trial demonstrated that during a 12-month follow-up after tooth extraction from the molar and premolar area, the width of the alveolar ridge was reduced by 50% (5–7 mm). 8

Immediate implant placement in molar extraction sockets may result in a high survival rate and minimal marginal bone loss. 2 However, immediate implant placement involves specific prerequisites. Success in this procedure relies on having optimal extraction socket conditions and a deep understanding of the local anatomy. 9 Moreover, sufficient bone quality and quantity are crucial for the viability of immediate implant placement as a treatment option. 10 11 A previous study showed that compared with the mandible, which contains dense alveolar cortical bone, the maxillary bone has a lower bone density. Specifically, the posterior maxilla has the lowest bone density after the tuberosity. 12 In addition, the presence of the maxillary sinus in the posterior maxillary region might limit the vertical height of the posterior maxilla. 13 Therefore, sinus floor elevation with or without a bone graft might be needed for immediate implant placement. 14 15

Several clinical studies measured alveolar and palatal bone thickness on cone beam computed tomography (CBCT) images. 16 17 18 19 A study measured anterior maxillary bone thickness and crestal bone height in the Saudi population using CBCT and found that males have greater facial bone thickness. 18 However, to our knowledge, no study has been conducted that measures the height, width, and quality of posterior maxillary alveolar bones in Saudi Arabia. Therefore, this retrospective study aimed to assess the height, width, and density of posterior maxillary bones in Saudi adults' molar dentulous areas using CBCT and compare these dimensions in males and females.

## Materials and Methods

This retrospective study was approved by the internal review board KFU-REC-2021-DEC-EA000322. The sample size was calculated using G*Power version 3.1.9 (Heinrich-Heine-Universität Düsseldorf, Düsseldorf, Germany); for an effect size of 0.75 mm, α = 0.05, and β = 0.9, the total sample size was 92. The sample consisted of the CBCT records of 102 patients randomly selected from a pool of patients who visited the dental clinic's complex at King Faisal University, Al-Ahsa, Saudi Arabia, between 2018 and 2022. The sample was divided into a male group consisting of 62 patients with a mean age of 29.92 ± 9.04 years and a female group consisting of 40 patients with a mean age of 29.70 ± 9.54 years.

The inclusion criteria were age ranging between 18 and 60 years and the presence of all permanent posterior teeth except the third molars on each side, with no or minimal bone loss. The exclusion criteria were the presence of any of the following: a molar with root canal treatment, a supraeruption, fused roots, or a periapical lesion.

CBCT images were captured with I-CT Vision QTM Version 1.9.3.14. (Imaging Sciences International, Hatfield, Pennsylvania, United States). The field of view was 130 × 160 mm with a voxel size of 0.25 mm, 120 kV, and 5 mA, with an exposure time of 2 to 7 seconds. The three-dimensional reconstruction and measurement of CBCT images were performed using BlueSkyPlan (Version 4.7.55, GmbH, Langenhagen, Germany).

### Variable Measurement

**Fig. 1:**
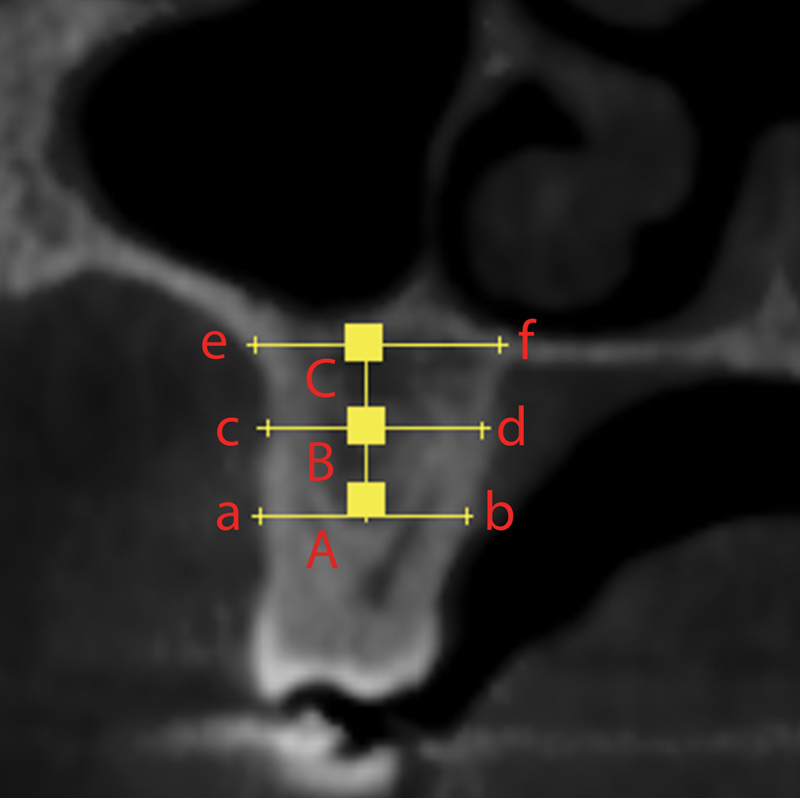
Linear measurements and density of posterior maxillary bone. ab, coronal width at the crest of interradicular bone; cd, middle width at midpoint between floor of maxillary sinus and the crest of interradicular bone; ef, apical width at the floor of maxillary sinus; A, midpoint of ab line, at which coronal density was recorded; B, midpoint of cd line, at which middle density was recorded; C, midpoint of ef line, at which apical density was recorded.

On the coronal view of the multiplanar reformation, seven variables (four distances and three densities) were measured at the central slice of each molar. The vertical height of the alveolar process was measured from the furcation area to the floor of the sinus. The horizontal width of the alveolar process was measured at the furcation area, at the floor of the sinus, and at the midway between both lines. The alveolar bone density was measured in the Hounsfield unit (HU) at the center of each horizontal line ( Fig. 1 ). Ten cases were randomly selected for remeasurement 1 month after the first measurement for reliability assessment. The data were assisted using an intraclass correlation coefficient (ICC), and each variable showed good reliability (ICC > 0.8)

### Statistical Analysis

Statistical analysis was performed using IBM SPSS Statistics for Mac, version 28 (IBM Corp., Armonk, New York, United States). Shapiro–Wilk's test was applied to ensure the normality of the variables. The right and left sides were compared using a paired sample *t* -test. Since there were no significant differences between the sides, the cases were combined for further analysis to assess the differences according to gender using multivariate analysis of variance (MANOVA). Density variables were not following the normal distribution; therefore, Mann–Whitney's *U* test was applied to assess the gender dimorphism in alveolar bone density. The level of significance was set at *p* < 0.05. Bonferroni correction for multiple corrections was applied when required and set at *p* < 0.008.

## Results

### Width and Height of Alveolar Bone at First Maxillary Molar Region

In all CBCT scans examined, the total number of first molar was 178 teeth with 105 (59%) of them for males and 73 (41%) for females. Regarding width measurements, the widest was the apical width at 14.68 ± 2.0 mm followed by the middle width at 13.99 ± 1.5 mm, and the smallest was the coronal width at 13.65 ± 1.3 mm. The overall measurement of height was 7.93 ± 3.8 mm, with more bone height in females (8.59 ± 3.5 mm) compared with males (7.74 ± 3.9 mm). MANOVA test showed that there was a significant difference in coronal ( *p* < 0.001) and middle ( *p* = 0.002) widths between males and females. However, there were no significant differences in the apical width and the bone height ( Table 1 ).

**Table 1:** Comparison of alveolar bone widths and height at posterior maxillary region between genders

	Male		Female		*p* -Value
	Mean	SD	Mean	SD	
First molar					
Coronal width	13.95	1.31	13.22	1.15	<0.001
Middle width	14.28	1.43	13.57	1.47	0.002
Apical width	14.51	1.91	14.93	2.05	0.168
Height	7.47	3.97	8.59	3.52	0.055
Second molar					
Coronal width	14.66	1.63	13.54	1.51	<0.001
Middle width	14.35	1.82	13.25	1.52	<0.001
Apical width	14.31	1.60	13.75	1.87	0.048
Height	7.29	3.00	8.66	3.16	0.007

Abbreviation: SD, standard deviation.

Note: Multivariate analysis of variance. Significance level was set at *p* < 0.05.

### Width and Height of Alveolar Bone at Second Maxillary Molar Region

Concerning the second molar, the overall number of teeth was 158 with 97 (61%) for males and 61 (39%) for females. In general, the alveolar bone height was 7.82 ± 3.1 mm, which was found to be greater in females (8.66 ± 3.16 mm) than in males (7.29 ± 3.00 mm). According to the widths of the bone at the second molar, the greatest was the coronal width at 14.22 ± 1.67 mm followed by the apical width at 14.09 ± 1.73 mm, and the smallest was the middle width at 13.93 ± 1.79 mm. Generally, a comparison between genders using MANOVA showed that males had greater coronal and middle widths than females ( *p* < 0.001); however, females had greater bone height than males ( *p* = 0.007) ( Table 1 ).

### The Density of Alveolar Bone at First Maxillary Molar Region

The overall bone density at the first molar region ranged between 218.53 ± 146.56 and 449.76 ± 154.79 HU. Regarding gender, coronal region density was 405.37 ± 144.69 HU in males and 449.76 ± 154.79 HU in females. Middle region density was 218.53 ± 146.56 HU in males and 245.24 ± 180.62 HU in females. In addition, apical region density was 274.94 ± 169.50 HU in males and 331.85 ± 148.50 HU in females. Mann–Whitney's *U* test showed no significant differences in coronal, middle, and apical densities according to gender ( Table 2 ).

**Table 2:** Comparison of alveolar bone density at posterior maxillary region between genders

	Male		Female		*p* -Value
	Mean	SD	Mean	SD	
First molar					
Coronal density	405.37	144.69	449.76	154.79	0.217
Middle density	218.53	146.56	245.24	180.62	0.370
Apical density	274.94	169.50	331.85	148.50	0.021
Second molar					
Coronal density	398.03	168.80	454.64	193.91	0.226
Middle density	222.53	141.56	238.43	165.32	0.657
Apical density	256.84	158.99	268.70	182.50	0.122

Abbreviation: SD, standard deviation.

Note: Mann–-Whitney's *U* test. Bonferroni correction for multiple comparison. Significance level was set at *p* < 0.008.

### The Density of Alveolar Bone at the Second Maxillary Molar Region

According to the density at the second maxillary molar region, the bone density ranged between 222.53 ± 141.56 and 454.64 ± 193.91 HU. Regarding gender, the coronal region density was 398.03 ± 168.80 HU in males and 454.64 ± 193.91 HU in females. The middle region density was 222.53 ± 141.56 HU in males and 238.43 ± 165.32 HU in females. Apical region density was 256.84 ± 158.99 HU in males and 268.70 ± 182.50 HU in females. Mann–Whitney's *U* test showed no significant differences in coronal, middle, and apical densities according to gender ( Table 2 ).

## Discussion

The growing preference for immediate implants among clinicians is driven by patients' desire for shorter rehabilitation periods. Additionally, immediate implant placement can mitigate the sequence of adaptive changes in both the horizontal and vertical dimensions of the alveolar bone and surrounding soft tissue following extraction. 20 This contributes to the preservation of socket integrity. 20 In light of this context, the main objective of the present study was to measure and evaluate the posterior maxillary alveolar bone width, height, and density specifically within the dentulous region of the first and second molars in both male and female patients who exhibited normal alveolar bone conditions.

Immediate implant placement in the posterior area of the maxilla presents unique challenges due to the presence of complex anatomical structures such as the maxillary sinus, socket width, multiple roots, and the risk of socket wall damage. 21 Additionally, the maxilla predominantly consists of spongy bone, making it the least dense bone composition and more challenging for immediate implant placement. 21

The measurement of alveolar bone height plays a crucial role in attaining primary stability. 22 However, the presence of a maxillary sinus might limit the bone height in the posterior maxillary alveolar bone. Mustakim et al classified that alveolar bone height measurement more than 8.0 mm provides sufficient vertical space to accommodate the implant without requiring sinus lifting. 22 Generally, when the alveolar bone height measures less than 6.0 mm, sinus lifting becomes mandatory. 22

In the present study, the bone height at first molar was 7.47 ± 3.97 mm in males and 8.59 ± 3.52 mm in females with no significant difference in dentulous patients. Similarly, Choi et al found the bone height at first molar was 5.38 ± 3.00 mm in males and 5.55 ± 3.04 mm in females with no significant gender dimorphism. 23 In addition, in our study, the alveolar bone height at the second molar was 7.29 ± 3.00 mm in males and 8.66 ± 3.166 mm in females with a significant difference. Likewise, Choi et al revealed that females had a significantly greater alveolar bone height than males at the second molar region. Despite this agreement with our results in the trend of the differences between genders, the differences in the height between the two studies might be due to ethnicity. 23 Meanwhile, Demirkol and Demirkol found the height of posterior maxillary bone in the dentate region to be 9.40 ± 4.24 mm. This difference from our results might be due to the different methodology applied and the ethnic group of the sample. 24

In our result, the coronal width of the alveolar bone at the first molar region was 13.65 ± 1.30 mm. Similarly, Cho et al 25 showed that this width was 12.38 mm at the first molar region in a South Korean population.

After tooth extraction, alveolar bone loss is expected. This was presented in the study of De Elío Oliveros et al 26 that assessed the dimensions of the posterior maxillary edentulous region. They found the apical width to be 11.05 ± 2.75 mm, middle width 9.04 ± 1.77 mm, and coronal width 7.32 ± 1.65 mm which agrees with a systematic review by Stumbras et al. 27 Compared with our study results, these widths are considerably smaller than those in our study, and this was because their measurements were performed on edentulous bone, whereas our sample was dentulous alveolar bone.

Additionally, CBCT images can be used to measure the bone volume and to quantitatively assess alveolar bone quality. 28 Nevertheless, the density of the bone plays a crucial role in immediate implant placement as it directly contributes to enhancing the primary stability of the implant. 29

Considering the bone density, Misch 30 classified five categories as follows: D1 bone had density >1,250 HU; D2, 850 to 1,250 HU; D3, 350 to 850 HU; D4, 150 to 350; and D5, <150 HU. In our results, the bone densities at the posterior maxillary alveolar bone ranged between 218.53 and 454.64 HU which falls between D4 (150–350 HU) and D3 (350–850 HU). Morar et al also found that the first maxillary molar positioned 2 mm from the alveolar crest exhibited a similar average value of 557.45 ± 275.61 HU in alveolar bone density. 31 This finding aligns with our results, particularly in terms of the coronal density of the first maxillary molar, which was 405.37 ± 144.69 HU in males and 449.76 ± 154.79 HU in females.

To our knowledge, this is the first study to measure the posterior maxillary alveolar bone quality and quantity in Saudi Arabia. However, this study might have been conducted on a limited sample size and geographical area. Future studies for measuring the bone quality and quantity in adult patients in the region are recommended for treatment planning of immediate implant placement. According to the results of this study, the average bone height had a relatively large standard deviation (3.9) suggesting a wide range of bone heights among the groups. Interestingly, most of these heights were lesser than the recommended bone height for placement of immediate implants. Because of that, CBCT scans might be essential before immediate implant placement for each patient for case-by-case assessment.

## Conclusion

In the current study, the minimum average alveolar bone height at the second maxillary molar region was 7.29 ± 30 mm with significant gender dimorphism. In addition, the minimum average alveolar bone density at the first maxillary molar region was 218.53 HU (D4) with no significant gender dimorphism. The anatomical information provided for the posterior maxillary alveolar bone region can be helpful for clinicians during treatment planning of immediate implant. CBCT radiograph might be recommended before immediate implant placement.

## References

[1] Cosyn J De Lat L Seyssens L Doornewaard R Deschepper E Vervaeke S The effectiveness of immediate implant placement for single tooth replacement compared to delayed implant placement: a systematic review and meta-analysis J Clin Periodontol 2019 46 21 224 241 30624808 10.1111/jcpe.13054

[2] Ragucci G M Elnayef B Criado-Cámara E Del Amo F SL Hernández-Alfaro F Immediate implant placement in molar extraction sockets: a systematic review and meta-analysis Int J Implant Dent 2020 6 01 40 32770283 10.1186/s40729-020-00235-5 PMC7413966

[3] Kan J YK Rungcharassaeng K Deflorian M Weinstein T Wang H L Testori T Immediate implant placement and provisionalization of maxillary anterior single implants Periodontol 2000 2018 77 01 197 212 29478284 10.1111/prd.12212

[4] Trombelli L Farina R Marzola A Bozzi L Liljenberg B Lindhe J Modeling and remodeling of human extraction sockets J Clin Periodontol 2008 35 07 630 639 18498382 10.1111/j.1600-051X.2008.01246.x

[5] Barone A Ricci M Tonelli P Santini S Covani U Tissue changes of extraction sockets in humans: a comparison of spontaneous healing vs. ridge preservation with secondary soft tissue healing Clin Oral Implants Res 2013 24 11 1231 1237 22784417 10.1111/j.1600-0501.2012.02535.x

[6] Zhang Y Ruan Z Shen M Clinical effect of platelet-rich fibrin on the preservation of the alveolar ridge following tooth extraction Exp Ther Med 2018 15 03 2277 2286 29456635 10.3892/etm.2018.5696 PMC5795808

[7] Ten Heggeler J MAG Slot D E Van der Weijden G A Effect of socket preservation therapies following tooth extraction in non-molar regions in humans: a systematic review Clin Oral Implants Res 2011 22 08 779 788 21091540 10.1111/j.1600-0501.2010.02064.x

[8] Schropp L Wenzel A Kostopoulos L Karring T Bone healing and soft tissue contour changes following single-tooth extraction: a clinical and radiographic 12-month prospective study Int J Periodontics Restorative Dent 2003 23 04 313 323 12956475

[9] Javaid M A Khurshid Z Zafar M S Najeeb S Immediate implants: clinical guidelines for esthetic outcomes Dent J 2016 4 02 21 10.3390/dj4020021 PMC5851264 29563463

[10] Rues S Schmitter M Kappel S Sonntag R Kretzer J P Nadorf J Effect of bone quality and quantity on the primary stability of dental implants in a simulated bicortical placement Clin Oral Investig 2021 25 03 1265 1272 10.1007/s00784-020-03432-z PMC7878229 32651646

[11] Buser D Chappuis V Belser U C Chen S Implant placement post extraction in esthetic single tooth sites: when immediate, when early, when late? Periodontol 2000 2017 73 01 84 102 28000278 10.1111/prd.12170

[12] Norton M R Gamble C Bone classification: an objective scale of bone density using the computerized tomography scan Clin Oral Implants Res 2001 12 01 79 84 11168274 10.1034/j.1600-0501.2001.012001079.x

[13] Whyte A Boeddinghaus R The maxillary sinus: physiology, development and imaging anatomy Dentomaxillofac Radiol 2019 48 08 2.0190205E7 10.1259/dmfr.20190205 PMC6951102 31386556

[14] Moraschini V Uzeda M G Sartoretto S C Calasans-Maia M D Maxillary sinus floor elevation with simultaneous implant placement without grafting materials: a systematic review and meta-analysis Int J Oral Maxillofac Implants 2017 46 05 636 647 10.1016/j.ijom.2017.01.021 28254402

[15] Liu H Liu R Wang M Yang J Immediate implant placement combined with maxillary sinus floor elevation utilizing the transalveolar approach and nonsubmerged healing for failing teeth in the maxillary molar area: a randomized controlled trial clinical study with one-year follow-up Clin Implant Dent Relat Res 2019 21 03 462 472 31044510 10.1111/cid.12783

[16] Sun Z Smith T Kortam S Kim D G Tee B C Fields H Effect of bone thickness on alveolar bone-height measurements from cone-beam computed tomography images Am J Orthod Dentofacial Orthop 2011 139 02 e117 e127 21300222 10.1016/j.ajodo.2010.08.016

[17] Zhang X Li Y Ge Z Zhao H Miao L Pan Y The dimension and morphology of alveolar bone at maxillary anterior teeth in periodontitis: a retrospective analysis-using CBCT Int J Oral Sci 2020 12 01 4 31932579 10.1038/s41368-019-0071-0 PMC6957679

[18] Sheerah H Othman B Jaafar A Alsharif A Alveolar bone plate measurements of maxillary anterior teeth: a retrospective cone beam computed tomography study, AlMadianh, Saudi Arabia Saudi Dent J 2019 31 04 437 444 31700220 10.1016/j.sdentj.2019.04.007 PMC6823811

[19] Ryu J H Park J H Vu Thi Thu T Bayome M Kim Y Kook Y A Palatal bone thickness compared with cone-beam computed tomography in adolescents and adults for mini-implant placement Am J Orthod Dentofacial Orthop 2012 142 02 207 212 22858330 10.1016/j.ajodo.2012.03.027

[20] Ravinder R Dubey P Raj S Mishra P Rajput A Immediate implant placement in posterior maxilla: a prospective clinical study Journal of Osseointegration 2021 13 04 185 190

[21] Thirunavakarasu R Arun M Abhinav R P Ganesh B S Commonly Used Implant Dimensions in the Posterior Maxilla-A Retrospective Study Journal of Long-Term Effects of Medical Implants 2022 32 01 25 32 35377991 10.1615/JLongTermEffMedImplants.2021038617

[22] Mustakim K R Eo M Y Lee J Y Myoung H Seo M H Kim S M Guidance and rationale for the immediate implant placement in the maxillary molar J Korean Assoc Oral Maxillofac Surg 2023 49 01 30 42 36859373 10.5125/jkaoms.2023.49.1.30 PMC9985995

[23] Choi Y J Kim Y H Han S S Alveolar bone height according to the anatomical relationship between the maxillary molar and sinus J Periodontal Implant Sci 2020 50 01 38 47 32128272 10.5051/jpis.2020.50.1.38 PMC7040445

[24] Demirkol M Demirkol N The effects of posterior alveolar bone height on the height of maxillary sinus septa Surg Radiol Anat 2019 41 09 1003 1009 31250139 10.1007/s00276-019-02271-2

[25] Cho H J Jeon J Y Ahn S J The preliminary study for three-dimensional alveolar bone morphologic characteristics for alveolar bone restoration Maxillofac Plast Reconstr Surg 2019 41 01 33 31531306 10.1186/s40902-019-0216-2 PMC6726725

[26] de Elío Oliveros J Del Canto Díaz A Del Canto Díaz M Orea C J Del Canto Pingarrón M Calvo J S Alveolar bone density and width affect primary implant stability J Oral Implantol 2020 46 04 389 395 32221558 10.1563/aaid-joi-D-19-00028

[27] Stumbras A Kuliesius P Januzis G Juodzbalys G Alveolar ridge preservation after tooth extraction using different bone graft materials and autologous platelet concentrates: a systematic review J Oral Maxillofac Res 2019 10 01 e2 10.5037/jomr.2019.10102 PMC6498816 31069040

[28] Van Dessel J Nicolielo L FP Huang Y Accuracy and reliability of different cone beam computed tomography (CBCT) devices for structural analysis of alveolar bone in comparison with multislice CT and micro-CT Eur J Oral Implantology 2017 10 01 95 105 28327698

[29] Merheb J Vercruyssen M Coucke W Quirynen M Relationship of implant stability and bone density derived from computerized tomography images Clin Implant Dent Relat Res 2018 20 01 50 57 29277972 10.1111/cid.12579

[30] Misch C E Density of Bone: Effect in Treatment Planning, Surgical Approach, and Healing St Louis Mosby 1993

[31] Morar L Băciuț G Băciuț M Analysis of CBCT bone density using the Hounsfield scale Prosthesis 2022 4 03 414 423

